# The False positive problem of automatic bot detection in social science research

**DOI:** 10.1371/journal.pone.0241045

**Published:** 2020-10-22

**Authors:** Adrian Rauchfleisch, Jonas Kaiser

**Affiliations:** 1 Graduate Institute of Journalism, National Taiwan University, Taipei, Taiwan (R.O.C.); 2 Communication, Journalism, & Media Department, Suffolk University, Boston, Massachusetts, United States of America; 3 Berkman Klein Center, Harvard University, Cambridge, Massachusetts, United States of America; 4 Alexander von Humboldt Institute for Internet & Society, Berlin, Germany; Ca' Foscari University of Venice, ITALY

## Abstract

The identification of bots is an important and complicated task. The bot classifier *"Botometer"* was successfully introduced as a way to estimate the number of bots in a given list of accounts and, as a consequence, has been frequently used in academic publications. Given its relevance for academic research and our understanding of the presence of automated accounts in any given Twitter discourse, we are interested in *Botometer*’s diagnostic ability over time. To do so, we collected the *Botometer* scores for five datasets (three verified as bots, two verified as human; n = 4,134) in two languages (English/German) over three months. We show that the *Botometer* scores are imprecise when it comes to estimating bots; especially in a different language. We further show in an analysis of *Botometer* scores over time that *Botometer*'s thresholds, even when used very conservatively, are prone to variance, which, in turn, will lead to false negatives (i.e., bots being classified as humans) and false positives (i.e., humans being classified as bots). This has immediate consequences for academic research as most studies in social science using the tool will unknowingly count a high number of human users as bots and vice versa. We conclude our study with a discussion about how computational social scientists should evaluate machine learning systems that are developed for identifying bots.

## Introduction

Identifying bots (here understood as fully automated accounts) on social media platforms like Twitter is a task that is as important as it is challenging. Indeed, being able to estimate the authenticity of a Twitter discourse by detecting malicious botnets or identifying how many “real” (i.e. human) followers a politician has, is an important baseline for computational social scientists. This is not only relevant in the context of political communication but especially so when it comes to the detection of disinformation campaigns and strengthening a platform’s security. Consequently, several methods have been introduced to get a handle on this problem, including a generic activity filter [1], an analysis of posting patterns [2], as well as machine-learning based classifiers [3]. Especially the trained classifier *Botometer* [3, 4] has established itself in the social sciences to estimate the number of bots within a given dataset.

In April 2018, for example, Pew Research Center published a large-scale study analyzing Tweets with links to popular websites finding that two-thirds of the analyzed tweets are posted by automated accounts [5]. Another study by Bessi and Ferrara [6] estimated that 19% of the tweets published during the 2016 presidential election in the US were posted by “social bots”. A study conducted in the German context found that 9.9% of the followers of German national party accounts on Twitter are “social bots” [7]. And a study published in Science found that fake news spread faster on Twitter due to humans and not bots [8]. These studies share a common feature: the bot detection tool *Botometer* [4] which was formerly known as *BotOrNot* [3]. It is, however, worth noting that these are by far not the only studies making use of this particular form of bot detection method. A simple Google Scholar search for ““botometer” OR “botornot”” yields 918 results (as of July 28, 2020) with 439 results since 2019. It is thus fair to say that the tool is arguably the most popular bot detection method in the social sciences and applications of the tools have been published both in field specific journals such as *Political Communication* [7] as well as high-ranked general-interest journals like *Science* [8] or *Nature Communications* [9]. This, then, would suggest that *Botometer* is the de-facto standard of bot detection in computational social science.

Bot detection, however, is notoriously tricky. Not only will bot creators (especially in the context of fraudulent social bots that pretend to be human) try to convince users as well as researchers applying their detection methods that their bots are legitimate humans but they will also adjust their bots to the newest identification methods and will thus often be one step ahead of researchers. This, of course, goes hand in hand with a certain uncertainty when it comes to detecting bots. There are numerous cases of Twitter accounts that look like bots but are actually human and vice versa; some accounts are both: semi-automated and semi-manual. To account for this complexity, Botometer does not say whether a user is a bot or not but rather will give a probability estimate between 0–1 (1 = most likely bot, 0 = most likely being human; the scale can also be 0–5 as shown on *Botometer*’s homepage). Researchers consequently have to define a manual threshold that seems reasonable to them if they want to classify accounts in a binary fashion. If an account has a higher score than the defined threshold, it will be defined as bot by the researchers. Wojcik et al. [5], for example, chose 0.43, while Keller and Klinger [7] opted for 0.76. In contrast, Zhang et al. [10] use a rather low threshold of .25 for the *complete automation probability* (CAP) which is also provided by *Botometer*.

For researchers, established tools like *Botometer* that come with a large body of peer-reviewed publications that lend authority and legitimacy to the tool as well as an easy to access API, are an important resource in their goal to shine a light into the role of bots on social media platforms such as Twitter and their potential impact on the public discourse. However, *Botometer* is not above criticism. Grimme et al. [11] show in their study, for example, that *Botometer* could not classify the hybrid and full automation bot accounts that the authors had created precisely. The original creators acknowledge these potential limitations of their tool and admit that “many machine learning algorithms, including those used by *Botometer*, do not make it easy to interpret their classifications” [12]. They further suggest in the official FAQ on the homepage of *Botometer* that “[i]t’s tempting to set some arbitrary threshold score and consider everything above that number a bot and everything below a human, but this is probably not the best way to think about it [the bot score]” [13]. Yet, as we have already indicated above, many studies relying on *Botometer* as a tool do use a fixed threshold [5, 7, 8].

Still, there are some notable few exceptions in the literature where researchers have validated existing tools like *Bototmeter* in a first step and, then, opted for a self-developed solution instead. While not explicitly discussing the diagnostic ability of *Botometer*, Fernquist et al. [14] indicate in their study of Swedish Twitter data that *Botometer* struggles with non-English language tweets and report that their own supervised classifier outperforms *Botometer*. Echeverría et al. [15] developed their own approach and showed that *Botometer* struggles with some of their bot data sets. Cresci and colleagues [16], for example, compare the performance of an earlier version of Botometer (BotOrNot) with other methods as well as their own method [17] and show that BotOrNot struggles with more recent versions of spambots. So while *Botometer* is one of the most popular bot detection some researchers have shown that other approaches outperform *Botometer* on certain data sets [16, 18, 19]. The examples above show that many different methods exist besides *Botometer*’s method. Orabi et al. [20] identified 53 different methods in their survey of the bot classification literature. In general, supervised and unsupervised methods can be distinguished [20] that are sometimes even combined [21]. Cresci [22] points out in his survey of the last decade of bot detection research that while the early days of bot detection methods were coined by supervised classifiers focusing on single accounts, more recently, many unsupervised methods [17, 23] focusing on groups [23] instead of single accounts were developed. Besides these mainly inferential approaches, Grimme and colleagues [11] discuss descriptive approaches in which researchers usually manually analyse specific campaigns. Some of these descriptive approaches are a combination of classifiers and manual analysis [24]. Even though many new bot detection methods are developed every year—outperforming *Botometer* in some cases—[15, 16, 18], we focus on *Botometer* as it is by far the most used tool in studies analyzing bots on Twitter [12], especially in social science [5, 7, 10].

In this paper we will use the term “bot” throughout. We acknowledge that other researchers have talked about “automated accounts” [1] or “social bots” [7] but since the creators of *Botometer* specifically talk about identifying “bots” we will use the same terminology [13]. It is noteworthy that the creators of *Botometer* will generally speak of “social bots” in their academic work [3, 6, 12, 25], but have not used the term in the tool’s FAQ system until recently. In Luceri et al. [26], one of the original *Botometer* creators even understands bot as short for “social media bot”, adding to the confusion. While there is agreement on the definition of “bot” (automation as a deciding factor), the social in “social bot” is contested. Yet, a debate about this part is beyond the scope of this paper and Gorwa and Guilbeault [27] have already clarified many aspects of the conceptual confusion in their bot typology.

The goal of this paper is twofold. Firstly, we want to critically assess, as we have shown above, one of the most popular bot detection tools used in social science. Secondly, we want to stimulate reflections on the role of computational methods as some of our findings help to better understand the limitations of machine learning systems that are developed to identify bots on social media platforms.

## Research questions

Given that it is very complicated to detect bots and *Botometer*’s prominence in the social sciences we ask: *How precise is Botometer in detecting bots*? More specifically we are interested in four different research questions. Firstly, we are interested in the general diagnostic ability of *Botometer*. We want to assess *Botometer* with the Receiver Operating Characteristics (ROC) curve as this is the common approach used, for example, by Varol et al. [4]. This approach allows us to assess how well *Botometer* can distinguish between bots and human users overall.

RQ1: How good is *Botometer*’s diagnostic ability for five distinct sets of Twitter accounts?

Yet, in practice many researchers analyze data sets that are sampled from the general Twitter population and manually define thresholds of what qualifies as a bot and what not. We are thus interested in the precision (percentage of accounts that are classified as bots) as well as the recall (percentage of identified bots from the whole population).

RQ2: How good is the precision and the recall of *Botometer* scores when used for five distinct sets of Twitter accounts representing the bot/human ratio in the general Twitter population?

As Fernquist et al.’s [14] analysis already indicates, there might be differences between languages with regard to *Botometer*‘s performance. The data analysis for the first two research questions allows us also to specifically assess whether there are differences between languages.

RQ3: Does *Botometer*’s performance differ between languages?

In an experimental setting, Grimme et al. [11] showed that the classification score is not stable over time. Therefore, we are interested in not only measuring the *Botometer* score once but instead tracking accounts over a longer period of time.

RQ4: How stable are *Botometer* classifications over time?

In answering these four questions, we want to add to the literature on bots by shining a light on some of the issues that *Botometer* has and discuss what consequences this has for social scientists conducting research on bots.

## Data and methods

To answer these research questions, we rely on five data sets. We included all official accounts of German members of parliament based on a list of personally confirmed official accounts (*German politicians*: n = 532) and all members of the 115th U.S. Congress with a Twitter account (*US politicians*: n = 516) [28] as “human” accounts. We also created a list of obvious bots (*new bots*: n = 935). We used all bots listed on https://botwiki.org/, a database with the goal to preserve interesting and creative Twitter bots as well as a number of other labelled bots we identified on Twitter. Most of these accounts are labelled as bots and for many even their code is available on GitHub. As most of these bots use the English language, we also collected an additional list of German language bots (*German bots*: n = 27). As a fifth dataset we use a manually annotated data set with human (n = 1747) and bot accounts (n = 826) that was created by the makers of *Botometer* [4]. We combined these data sets for our data analysis (see Table 1). For the first three research questions we need bots as well as human accounts combined as we are evaluating how well *Botometer* can distinguish automated from human accounts. We therefore combine the different datasets (see Table 1). For RQ4, however, we can use single datasets consisting only of bots or humans.

**Table 1:** Data sets and how we combined them for our analysis.

dataset	In our analysis	Valid accounts
German politicians	all/ German politicians and bots	human = 516
US politicians	all/ US politicians and bots	human = 502
New bots	all/ US politicians and bots/ German politicians and bots	bots = 928
German bots	all/ German politicians and German bots	bots = 27
Varol	all/ Varol et al.	bots = 699 / human = 1462

**Fig 1:**
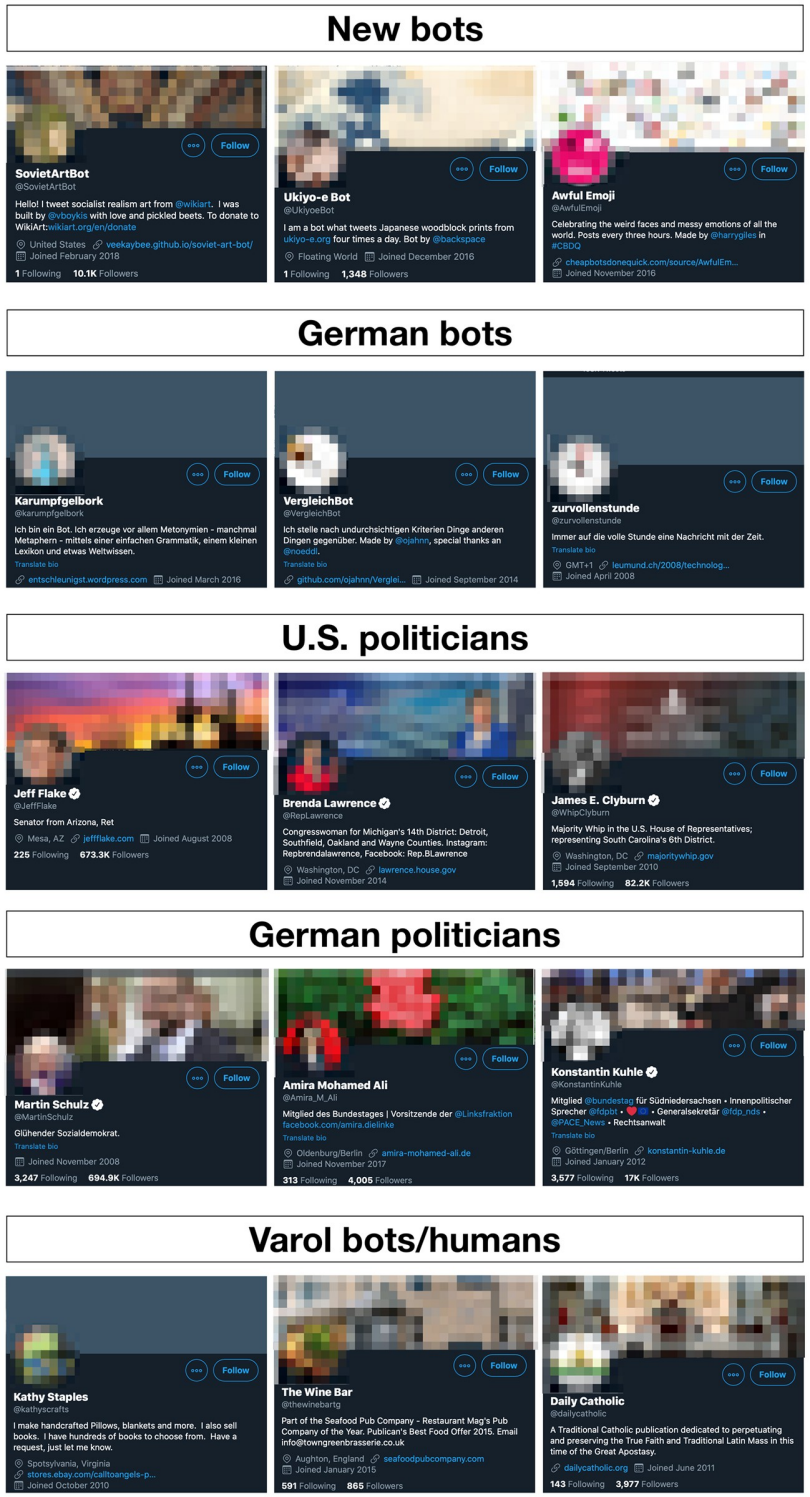
Example accounts for each data set.

Our selection of the data sets is based on one basic assumption: bot detection tools like *Botometer* will perform better in distinguishing between bots and humans when the cases are clear-cut. As can be seen in Fig 1, clear cut bot accounts will often outright state in their description that they are, in fact, bots; occasionally even sharing the code on GitHub. Similarly, clear-cut human cases will often have indicators that signal their authenticity such as a blue checkmark (Twitter’s verification badge) or their personal website. To make it even clearer, we chose elected national politicians with their official accounts, i.e. people that are in the public limelight and often have high follower numbers, where suspicious Twitter activity would automatically lead to media attention and increased scrutiny. In Germany, for example, journalists have discovered a network around AfD politicians’ Twitter accounts that consisted of sockpuppets that boosted engagement numbers [29]. This is not to say that politicians might not make use of any automation but rather that this form of automation is and should not be confused with bots or social bots. Finally, we also included Varol’s list that included both bots as well as humans. While we were not always able to individually verify whether an account was a bot or a human, we trust in the initial categorization which parts of *Botometer’s* training is based on. As a result, we have four data sets of accounts that human coders would have no difficulty in differentiating between bots and humans and one which was used to train the *Botometer* classifier on. Based on these criteria, we would expect the *Botometer* tool to perform rather well as the “real world” data from Twitter is much messier.

To conduct this test, we created a Python script that ran every day at 00:00 UTC time on an Ubuntu Linux server for 3 months (3rd March– 2nd of June, 2019). The data collection went uninterrupted. For every account in our sample (n = 4,583) we requested the Twitter data via the Twitter search API and the *Botometer* user scores via the *Botometer* API giving us a total of 374,724 valid scores. For the analysis we accessed Botometer v3 [12] which was the latest version of the tool in March 2019. Besides an empty timeline (*Botometer* needs at least one tweet– 2,021 calls had to be excluded), missing accounts (mainly from Varol’s data set which includes accounts that do not exist anymore– 11,197 calls) and private accounts (also mainly from Varol’s data set– 33,608 calls had to be excluded), only 86 calls (less than 0.05% of all calls) returned an error because of a technical API issue from *Botometer* or the Twitter API. Eventually we could analyze 4,134 existing accounts that could be accessed at least once during the process of data collection and have at least one tweet on their timeline (see Table 1). For the following analysis we use the language-independent universal *Botometer* score between 0 and 1 (which corresponds to the score 0–5 also used by *Botometer*—e.g., 0.5 will be 2.5), the specific English language Botometer score between 0 and 1, as well as *Botometer*’s newly integrated English and universal complete automation probability (CAP) (ranging between 0 and 1). While we are aware that the *Botometer* creators offer more fine-grained feature scores besides the overall universal scores, we focus on the universal overall scores as they are the scores used by social scientists in their studies and are independent from the language that the accounts tweet in. Still, as we are interested in language differences we also report *Botometer*’s English scores for our third research question (see also S1–S5 Figs for all other visualizations with the English score and CAP).

For RQ1 we compared the ROC-AUC (Receiver Operating Characteristics-Area Under The Curve) with the old ROC-AUC reported in older studies for *Botometer* to evaluate *Botometer*’s diagnostic ability. ROC-AUC indicates how well a model discriminates between two classes (bot and no bot). The true positive rate on the y-axis is plotted against the false positive rate on the x-axis. While this statistic is useful to directly compare the overall performance of different models and labelled data sets with regard to the accuracy of the classifications, this evaluation method also has its limitations [30, 31]. Researchers, for example, are often not interested in all potential score thresholds as used in the AUC-ROC approach and instead only in specific classification values [32]. The AUC-ROC approach does not consider the cost of false-positive and false-negatives and does not fully consider imbalances in the general population [33]. However, in their original paper for the first version of the tool (*BotOrNot*) the authors transparently warn that the accuracy of their model (they received a ROC-AUC of 0.95/0.94) overestimates future performance due to the age of the training data [3].

As the ROC-AUC approach has limitations, we also use a different approach for RQ2. In bot research the biggest problem is the lower occurrence of bots in relation to humans. In the general population of all Twitter users there are more real users than bots. However, the ROC-AUC approach does not consider the baseline in the Twitter population. This is a general problem with classifiers if the structure of the population in which the classifier should be later used is ignored. In the case of bots, we want to clearly identify bots and make statements about the number of bots in a data set. This becomes even clearer when the main goal as well as the wording in bot studies is considered. Researchers are interested in the number of bots and not mainly in the number of human users. Therefore, even a small false-positive rate (FPR) has a large impact on the results. The data used to test (as in our case) and train *Botometer* is often more balanced than the rather imbalanced general Twitter population. Saito and Rehmsmeier [33] advocate in such cases to evaluate the performance of a classifier based on the Precision-Recall (PR) curves as they “allow for an accurate and intuitive interpretation of practical classifier performance”. While the so-called Matthews correlation coefficient is better than the F1 score if researchers use only one specific threshold, PR curves are recommended [34, 35] if different threshold levels exist as in the case with *Botometer*. To simulate a population that represents the same assumed real ratio of the general Twitter population with 15% bots [4] and 85% non-bots, we resampled the different data sets for RQ2.

## Results

**Fig 2:**
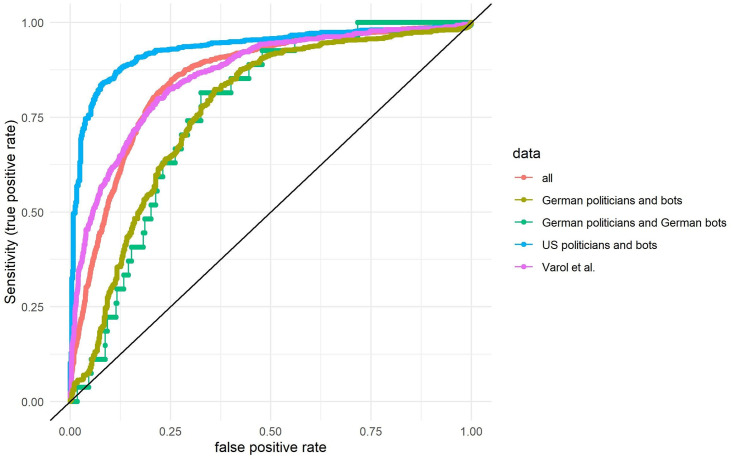
Receiver operating characteristics curve for Botometer and the universal score (average over 3 months for each account). The x-axis represents the false positive rate and the y-axis represents the true positive rate (sensitivity).

Our analysis (see Fig 2 and Table 2) shows that the ROC-AUC is worse with our complete data (AUC = 0.85) than with the data sets used in the *Botometer* creators’ original papers which received an AUC of 0.94 [4]. However, their baseline model also received only an AUC of 0.85 in the original paper. The nearer the ROC curve (Fig 2) is to the upper-left corner and the more space is under the curve, the better *Botometer* can distinguish between bots and humans. In the lower-left corner the curve starts with a threshold of 0 for the *Botometer* which means the false-positive rate is 0 but at the same time 0 of the bots in the population will be identified. The curve ends with a threshold of 1 in the upper-right corner as then all bots in the population will be identified. However, such a threshold also means that all human accounts will be wrongly classified as bots (false-positive rate = 1). Each point in the curve thus represents a specific threshold for which the true positive rate as well as the false positive rate is shown.

**Table 2:** ROC-AUC as well as the PR-AUC universal score.

Data set	ROC-AUC	ROC-AUC sample	PR-AUC	PR-AUC sample
All	0.85 (0.84–0.86)	0.85 (0.85–0.86)	0.77 (0.75–0.79)	0.50 (0.49–0.51)
German politicians and German bots	0.76 (0.70–0.83)	0.76 (0.76–0.77)	0.10 (0.07–0.14)	0.27 (0.27–0.27)
German politicians and bots	0.77 (0.74–0.79)	0.76 (0.76–0.77)	0.81 (0.78–0.84)	0.30 (0.30–0.31)
US politicians and bots	0.93 (0.92–0.94)	0.93 (0.93–0.93)	0.96 (0.95–0.97)	0.79 (0.79–0.80)
Varol et al.	0.86 (0.84–0.88)	0.86 (0.86–0.86)	0.76 (0.73–0.79)	0.58 (0.58–0.59)

ROC-AUC as well as the PR-AUC scores for the original data sets as well as the weighted resampled data sets (sample = 100,000) for the universal score. The 95% confidence intervals based on 10,000 stratified bootstrap replicates are shown in brackets.

When applying this to our data sets, we see that the classifier has the highest AUC score for the *US politicians and bots* data set (0.93) followed by Varol et al.’s labeled data set (0.86). The score for the *German politicians and German bots* yields a lower score (0.76). As we only had a few German bots we also tested the *German politicians and bots* (with the new bots instead of the German bots) which had a slightly higher AUC (0.78).

**Fig 3:**
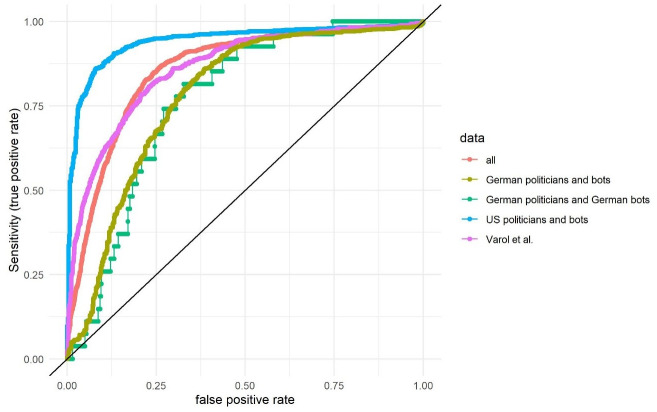
Receiver operating characteristics curve for Botometer and the universal complete automation probability (CAP) (average over 3 months for each account). The x-axis represents the false positive rate and the y-axis represents the true positive rate (sensitivity).

We, then, calculated the ROC-AUC for the universal CAP (complete automation probability; Fig 3 and Table 3). We could observe similar results as before for the universal score: the US politicians received the highest AUC (0.94) followed by Varol et al. (0.86), the complete data set (0.86), the German politicians with the new bots (0.78) and the German politicians and the German bots (0.77).

**Table 3:** ROC-AUC as well as the PR-AUC scores universal CAP.

Data set	ROC-AUC	ROC-AUC sample	PR-AUC	PR-AUC sample
All	0.86 (0.85–0.87)	0.86 (0.86–0.86)	0.78 (0.76–0.80)	0.51 (0.50–0.52)
German politicians and German bots	0.77 (0.70–0.84)	0.77 (0.76–0.77)	0.10 (0.08–0.14)	0.27 (0.27–0.27)
German politicians and bots	0.78 (0.75–0.81)	0.78 (0.77–0.78)	0.81 (0.79–0.84)	0.31 (0.30–0.31)
US politicians and bots	0.94 (0.93–0.95)	0.94 (0.94–0.94)	0.97 (0.96–0.98)	0.81 (0.81–0.82)
Varol et al.	0.86 (0.84–0.88)	0.86 (0.86–0.87)	0.76 (0.73–0.79)	0.58 (0.57–0.59)

ROC-AUC as well as the PR-AUC scores for the original data sets as well as the weighted resampled data sets (sample = 100,000) for the universal CAP. The 95% confidence intervals based on 10,000 stratified bootstrap replicates are shown in brackets.

For RQ2 we suggest a thought experiment based on the data sets analyzed in this study. While the data sets are not perfectly balanced, they definitely do not represent the imbalanced ratio of bots and human accounts of the real Twitter population. We thus assume for our simulation that the Twitter population has 15% bots [4] and 85% non-bots. We first gave every single account a sampling probability that reflects the occurrence of cases in the Twitter population (n bots/0.15 and n humans/0.85) and took 100,000 random samples with replacement for every analyzed data set. As a result, we created new data sets that have 15,000 bots and 85,000 non-bots. We then calculated for all the data sets the ROC-AUC as well as the PR-AUC (see Tables 2 and 3). As expected the ROC-AUC is the same with the new sample data for all analyzed data sets, which is also in line with what Saito & Rehmsmeier [33] show in their study. However, the PR-AUC shows a different score for the resampled data sets as the PR-AUC changes depending on the imbalance in a data set. The only exception is the in its original form highly skewed German politicians and German bots data set which shows an improvement but overall still an extremely low score (from 0.10 to 0.27).

**Fig 4:**
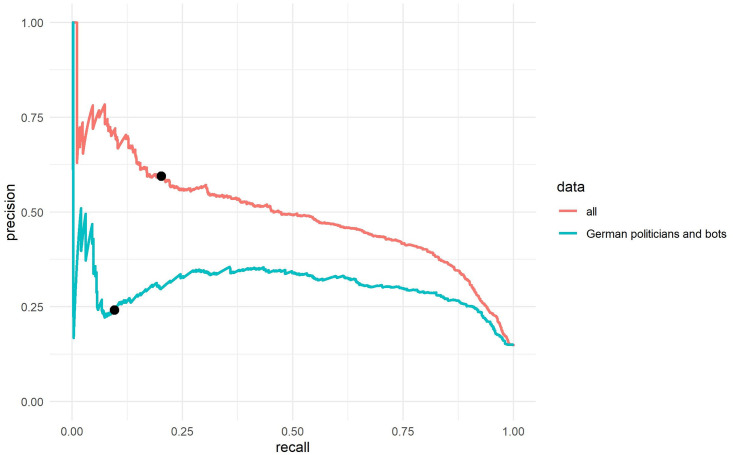
Universal score precision-recall curves for the resampled data sets. We consider the population baseline on Twitter (15% bots) for the universal Botometer score, black points indicate the precision and the recall for the Botometer score 0.76. With the German politicians and bots data set, for almost every threshold level the identified sample of bots has more humans than real bots (precision). The x-axis represents the recall (sensitivity) and the y-axis represents the precision.

The PR curves allow us to better evaluate how many bots will be amongst the accounts that *Botometer* classified as bots, and how many bots were not identified if we use a high threshold (as many studies do with this tool, e.g. [7]). PR curves start on the right with a *Botometer* score threshold of 0. If an extremely low threshold is used, all bots in the population will be identified (recall = 1) but the number of false-positive cases will be extremely high. The precision is thus .15 as 85% of the accounts in the sample are humans. On the left, the curve ends with an extremely high threshold for the *Botometer* score which means that the precision is extremely high (the classified bot accounts are all bots) but the recall is extremely low (almost all bots in the general population are not identified). We can now also check the precision and recall for specific thresholds. For example, if 0.76 is used as a threshold for the resampled *all* data set, 59% real bots (precision = 0.59) and 41% false-positive humans will be amongst the accounts classified as bots (see Fig 4). However, around 80% (recall = 0.2) of the bots in the population will not be identified (false-negatives). If we consider the *German politician and bots* data set, the results are even worse. Of the accounts classified as “bot” only around 24% (precision = 0.24) would be bots and 76% of the accounts would be false-positive human accounts. Moreover, around 90% (recall = 0.1) of the bots in the Twitter population would not be identified (see Fig 4).

**Fig 5:**
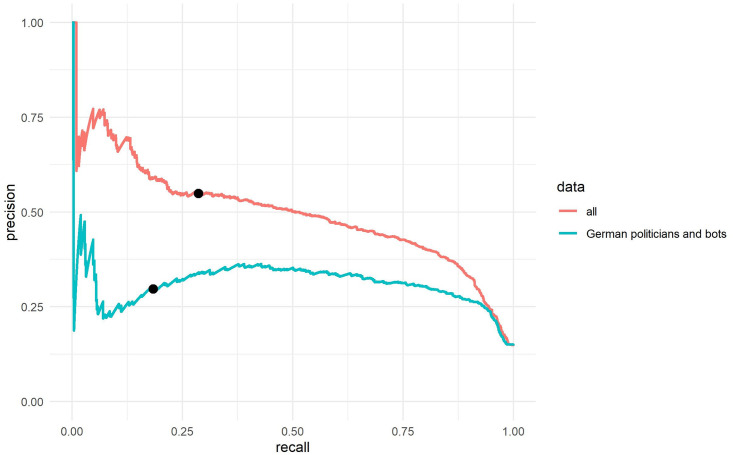
CAP precision-recall curves for the resampled data sets. We consider the population baseline on Twitter (15% bots) for the universal CAP Botometer score, Black points indicate the precision and the recall for the universal CAP 0.25. With the German politicians and bots for almost every threshold level the identified sample of bots has more humans than real bots (precision). The x-axis represents the recall (sensitivity) and the y-axis represents the precision.

We then also calculated the PR-AUC for the universal CAP with the newly weighted resamples (see Table 3). With regard to the PR-AUC and the ROC-AUC, the results are almost the same as for the universal *Botometer* scores. When calculating the CAP and using a threshold of 0.25–as used by Zhang et al. [10], the accounts, classified as bots by *Botometer*, would consist of around 55% bots (precision = 0.55) and 45% humans. However, around 71% (recall = .29) of the bots in the population would not be identified. If we calculate the same for the *German politicians with bots*, we have 70% false-positive human accounts and 30% bots (precision = 0.3) in the sample that *Botometer* classified as “bots” (see Fig 5). Around 82% of the bots (recall = 0.18) in the population would not be identified. For these tests, too, we used a resample of 100,000 accounts for every data set which are based on the population assumption that 15% of bots were present in the Twitter population. This quantitative thought experiment, then, shows the impact these scores can have on findings estimating the number of humans and bots.

In our third research question we are interested in the difference between languages. First of all, *Botometer*’s English score (Table 4) works better with English accounts (e.g., Varol et al. data set: ROC-AUC English = 0.90; universal = 0.86; PR-AUC sample English = 0.69; universal = 0.58) and performs worse with mainly non-English accounts (e.g., German politicians and German bots data set: ROC-AUC English = 0.69; universal = 0.76; PR-AUC sample English = 0.23; universal = 0.27). The same holds true for the universal and English CAP (Table 5). A comparison between the different data sets shows that the tool works better with data from the US/English (see Figs 2–5 –see also S1 and S3 Figs). However, *Botometer* is neither working well with the German politicians nor the new English-language bots we tested, even with the English scores. We further tested with unpaired DeLong tests [36, 37] whether the ROC-AUC of the German data sets are statistically different from other data sets. Our test shows that the ROC-AUC for the German data sets are significantly different from the ROC-AUC of the *US politicians and bots* with regard to the universal score (German politicians and bots: *D* = -10.58, df = 2133.1, *p* < 0.001; German politicians and German bots: *D* = -4.67, *df* = 586.12, *p* < 0.001; Bonferroni correction *α* = 0.005) as well as the universal CAP (German politicians and bots: *D* = -4.81, df = 579.12, *p* < 0.001; German politicians and German bots: *D* = -10.65, df = 2066.3, *p* < 0.001; Bonferroni correction *α* = 0.005; see also Tables 2 and 3). The same holds true for the English scores. Furthermore, if *Botometer* is used with German accounts, the real rate of false-positives is higher than researchers might assume when their only indicator for performance is the ROC-AUC. While the ROC-AUC approach is a good indicator for the original study and data set, researchers should be cautious to assume the same level of discriminatory power can be reached with a new data set from a different account population. Additionally, researchers should consider the absolute number of false-positives in the classified sample and report the MCC, precision as well as the recall for the threshold used in a study.

**Table 4:** ROC-AUC as well as the PR-AUC English score.

Data set	ROC-AUC	ROC-AUC sample	PR-AUC	PR-AUC sample
All	0.88 (0.86–0.89)	0.88 (0.87–0.88)	0.80 (0.79–0.82)	0.56 (0.55–0.56)
German politicians and German bots	0.68 (0.60–0.77)	0.69 (0.68–0.69)	0.08 (0.06–0.11)	0.23 (0.22–0.23)
German politicians and bots	0.82 (0.79–0.84)	0.82 (0.81–0.82)	0.83 (0.81–0.86)	0.34 (0.34–0.35)
US politicians and bots	0.93 (0.92–0.94)	0.93 (0.93–0.93)	0.96 (0.95–0.97)	0.75 (0.74–0.76)
Varol et al.	0.90 (0.88–0.91)	0.90 (0.90–0.90)	0.83 (0.80–0.85)	0.69 (0.68–0.70)

ROC-AUC as well as the PR-AUC scores for the original data sets as well as the weighted resampled data sets (sample = 100,000) for the English score. The 95% confidence intervals based on 10,000 stratified bootstrap replicates are shown in brackets.

**Table 5:** ROC-AUC as well as the PR-AUC for the English CAP.

Data set	ROC-AUC	ROC-AUC sample	PR-AUC	PR-AUC sample
All	0.88 (0.86–0.89)	0.87 (0.87–0.88)	0.80 (0.79–0.82)	0.55 (0.54–0.55)
German politicians and German bots	0.69 (0.60–0.78)	0.69 (0.69–0.70)	0.08 (0.06–0.11)	0.22 (0.22–0.22)
German politicians and bots	0.82 (0.79–0.84)	0.82 (0.81–0.82)	0.83 (0.80–0.86)	0.34 (0.33–0.34)
US politicians and bots	0.93 (0.92–0.95)	0.94 (0.93–0.94)	0.96 (0.95–0.97)	0.75 (0.75–0.76)
Varol et al.	0.90 (0.88–0.91)	0.89 (0.89–0.90)	0.82 (0.80–0.85)	0.68 (0.67–0.69)

ROC-AUC as well as the PR-AUC scores for the original data sets as well as the weighted resampled data sets (sample = 100,000) for the English CAP. The 95% confidence intervals based on 10,000 stratified bootstrap replicates are shown in brackets.

**Fig 6:**
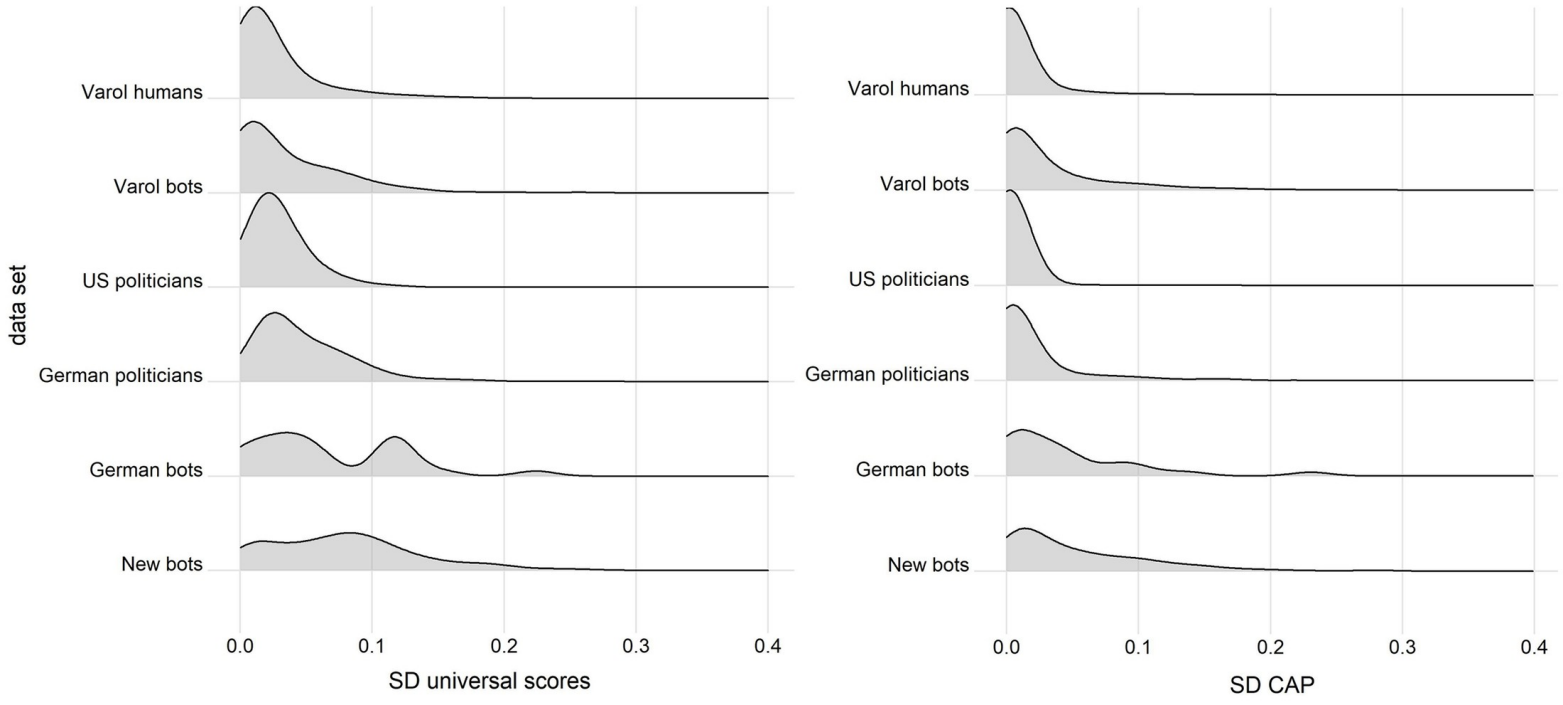
Density plot of the SDs for single accounts plotted for each group. Left for the Botometer universal score, right for the Botometer CAP. Bandwith of 0.015 was used for the CAP and the universal scores. The x-axis represents standard deviation and the y-axis represents the density.

Finally, in RQ4 we are interested in the temporal volatility of *Botometer* scores. We identified substantive changes in the bot scores on an individual level. We first calculated the standard deviation of the universal score as well as the universal CAP for every single account, ie. for each account in our analysis based on all individual measurements over time and then plotted the density of all the individual account SDs for every individual data set to evaluate the volatility of the scores over time. The SD will be high if the *Botometer* scores of an account have a high variability over time. Our analysis indicates that especially the accounts in the bot datasets show high standard deviations and thus are rather volatile over time (Fig 6). The only noteworthy difference between the universal score and the universal CAP is that the CAP overall seems to be slightly less volatile overall (see also S2 Fig).

Lastly, we also checked how many accounts in each data set are classified differently during the three months. The volatility alone is only a problem if single accounts are depending on the day below or above a chosen threshold used by researchers to classify bots. If we chose a threshold of 0.76, 27.2% of the new bots, 22.2% of the German bots and 13.9% of the Varol bots will have a daily measurement above and below the threshold at least once. For the humans the volatility is less severe with regard to the classification based on a high threshold. 7.4% of the German politicians, 3.1% of the Varol humans and only 0.6% of the US politicians have a daily measurement above and below the threshold at least once.

We also calculated the change of classification for a threshold of 0.25 for the universal CAP. We see that 37.5% of the new bots, 33.3% of the German bots, 10.7% of the German politicians as well as 1% of the US politicians have a score below as well as above the threshold at least once. Of the Varol bot data set 17.7% and of the Varol human data set 4.7% are a bot or a human at least once.

**Fig 7:**
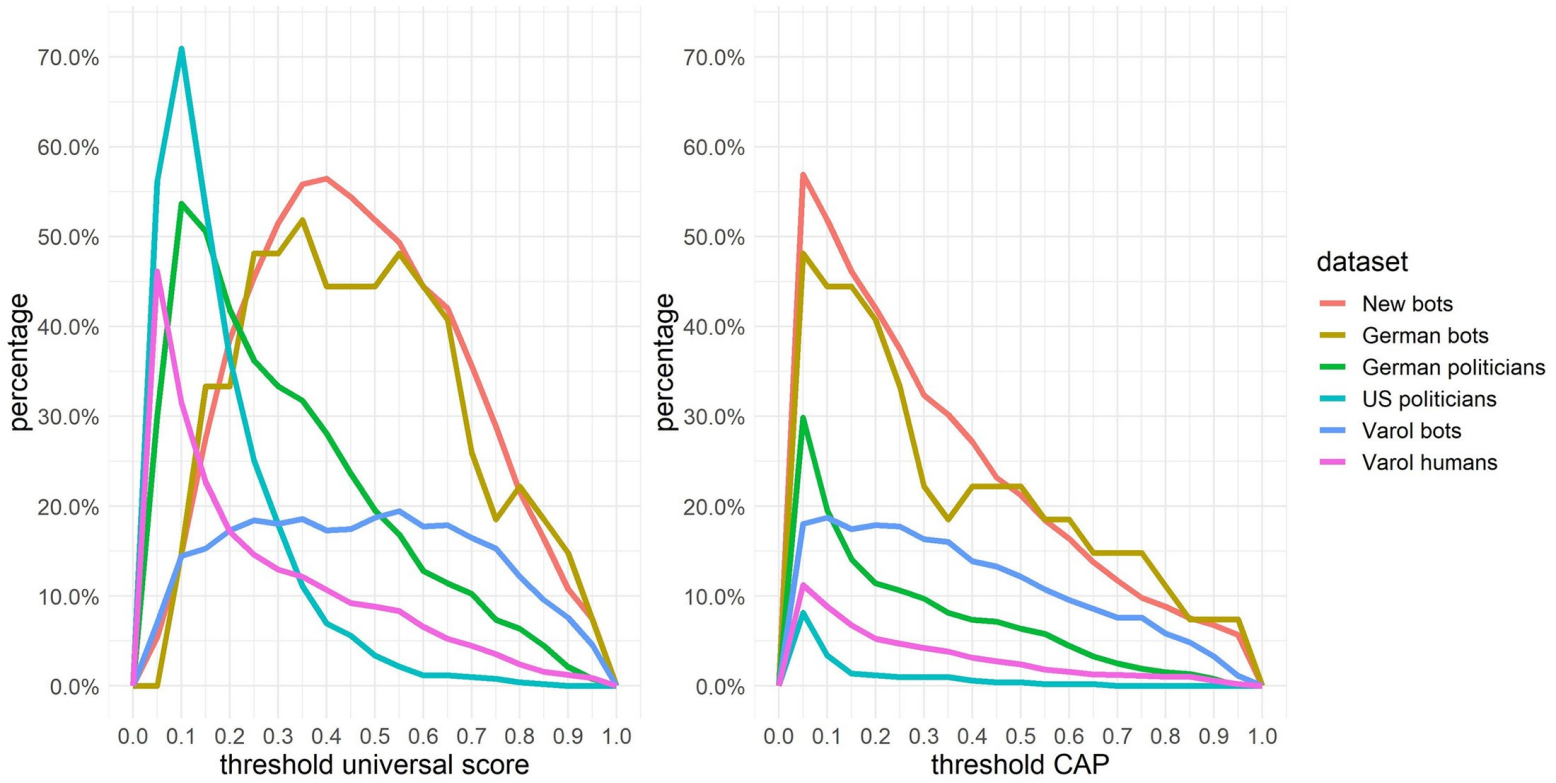
Changing score over time. Percentage of accounts (y-axis) that have at least once a score in the three months below as well as above the threshold for all thresholds between 0 and 1 in steps of 0.05. The x-axis represents the chosen threshold for the *Botometer* score. Left for the universal score, right for the universal CAP.

We additionally calculated the percentage of accounts that have a score in the three months below as well as above the threshold at least once for all thresholds between 0 and 1 in steps of 0.05 (see Fig 7; see also S3 Fig). Our analysis shows that overall bots are more prone to have a score below and above the threshold for both the universal CAP and the universal score at least once. However, the universal CAP, works better for the *new bots* as well as the *German bots*.

## Discussion

In a time where terms like “computational propaganda” [38], “disinformation campaigns” [39] or “coordinated inauthentic behavior” [40] are often used, identifying the automated accounts that spread disinformation or attempt to shift the conversation seems paramount. Indeed, being able to estimate the degree of inauthentic behaviors within a given online discourse and understanding their patterns could lead to the identification of coordinated disinformation campaigns. Consequently, there was and still is an academic demand for tools like *Botometer* that did not only come with an API but, more importantly, also with the necessary academic credentials (i.e. institution and citations). *Botometer* was and is a very good example of how supervised machine learning models analysing single accounts can be trained and what the potential outcome might be.

Yet, as we show in this study, researchers should be cautious using tools that were developed by other scientists, sometimes in a totally different context. A high ROC-AUC score with the original data set and research setting does not translate into a good “precision” for computational social science studies investigating a different context as we show for Botometer v3. Our results are thus in line with what some prior studies could show for older versions of *Botometer* [14, 16]. Even a good diagnostic ability (ROC-AUC) leads to problematic outcomes when the population baseline is considered. In other words: just because the model works with the test data set, does not mean that the results are reliable for a specific use case. As scientists we should care about reliability, validity, and reproducibility [41, 42]. In many of the social science studies that make use of *Botometer*, these criteria have been disregarded. As we have shown with our analysis, *Botometer* has problems in identifying false positives and false negatives in our collections when applying analytical approaches from computational social sciences; especially with thresholds used in prior studies. This is noteworthy, as our data sets consisted either of accounts that *Botometer* was directly trained on or where the cases were clear-cut and easily identifiable for a human coder. The *Botometer* creators [43] argue in their newest paper that some of the German politicians’ accounts used in our study might have a certain degree of automation. However, we manually checked all false-positive politician accounts (score above .76) and only one out of 27 false-positive politicians shows some behavior that might be classified by *Botometer* as Automation. Namely, the politician had synchronized her Facebook account with her Twitter account and all Facebook posts were also pushed over Twitter. This shows that *Botometer*’s misclassification is not due to real automation by the political accounts. Indeed, most of the other accounts that had been falsely classified as bots were mostly inactive and hadn’t tweeted in that time period. Furthermore, we show that the scores change over time, thus, effectively, making studies that use *Botometer* data from one day or a week hard to replicate. *Botometer* worked, unsurprisingly, better on accounts that the algorithm was trained on even in our analysis over time. However, the tool was imprecise over time for the new bots as well as the German dataset. In addition, bots that are not aimed at entertaining other users but rather aim at obfuscating their automated identity and real goals (e.g., spreading misinformation) will pretend to be humans, thus making it even harder for bot detection tools to identify them [11, 16]. In this sense, distinguishing clearly between humans as well as bots is the minimum that bot detection tools should accomplish. Indeed, as researchers we should be careful when relying on existing tools and, as our thought experiment highlighted, also have to be mindful of the population we apply the classifiers on. If the numbers of bots are in the minority for the general population (as we assume in this case on Twitter), even a small false-positive rate might lead to a high number of human accounts wrongly classified as bots.

**Fig 8:**
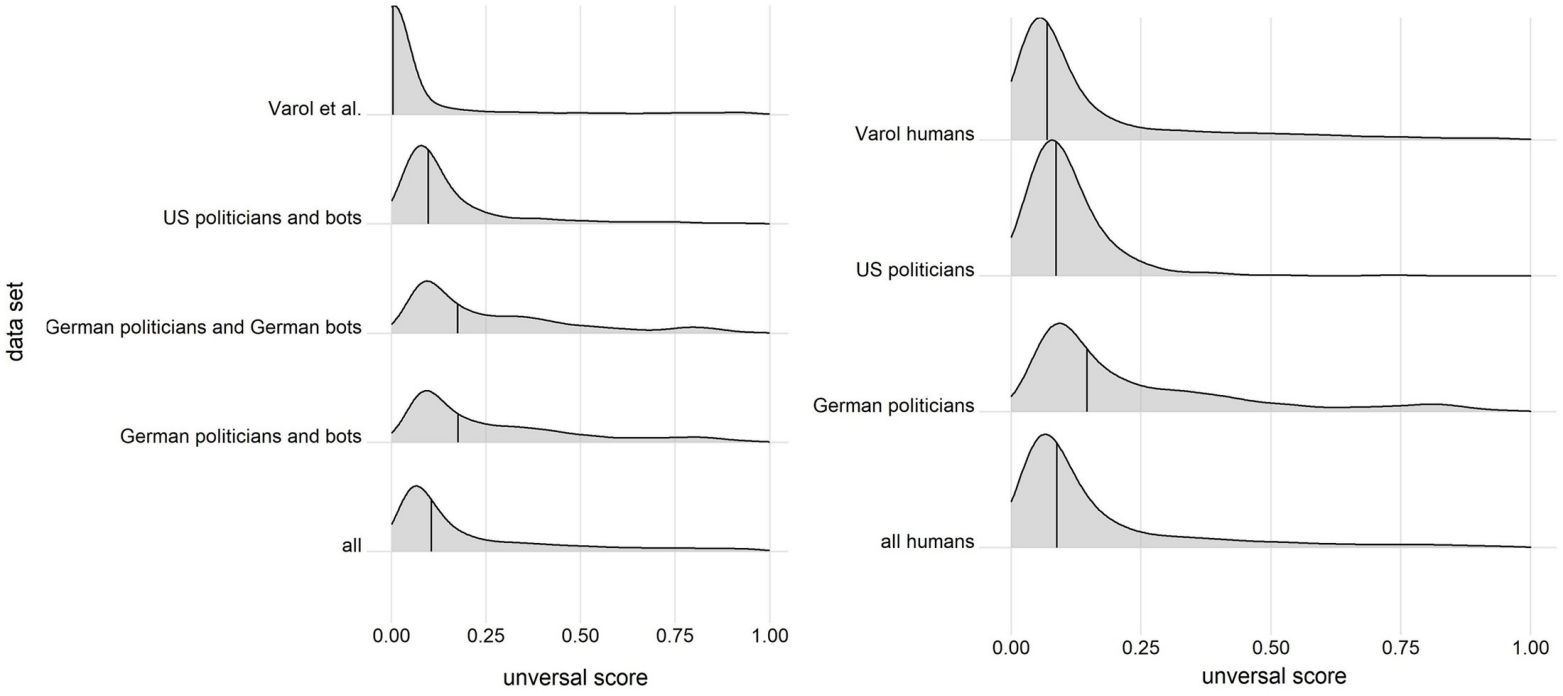
Density plots for the different data sets. Left: Density plots for the different combined data sets in our analysis showing the distribution of Botometer’s universal score. We used the resampled data sets with 15% bots and 85% humans with a total n = 100,000 for each data set. Right: Density plots for the human accounts data sets. Lines indicate the median, a bandwidth of 0.04 was used for all data sets. The x-axis represents the *Botometer* score and the y-axis represents the density.

This, then, raises the question if researchers should make use of *Botometer* at all. And while an argument in favor of *Botometer* would be that, while not perfect, it is at least an approximation towards identifying bots on Twitter, our counter-argument would be that the issue of false positive and false negative rates for specific thresholds with data sets based on the general Twitter population as well as the tool’s vulnerability to temporal changes or different languages do call the tool’s classifications into question. A solution researchers might come up with would be to shift the focus from thresholds to the overall distribution of the raw scores to evaluate the prevalence of bots in a data set, i.e. to measure the “bottiness”. However, based on our analysis we would not recommend such an approach as different data sets with the same human to bot ratio as well as our human data sets can look differently in density plots (see Fig 8; see also S5 Fig). While the shift from individual accounts analysed with supervised classifiers to unsupervised classifiers focusing on group behaviour is promising [16, 22], researchers should also be cautious if they arbitrarily choose thresholds [44]. Even a good classifier can be bad if researchers use it in the wrong context without any validation.

However, we also have to add as a limitation that we tested the tool with a few specific data sets. We have chosen data sets that make it easy for us to evaluate the ground truth and we still expect very similar results with future analyses. Furthermore, we have used a data set of bots that includes some of the most obvious bots. If researchers manually validate the tool again with their own unique data analysis, they of course should make use of the tool. Our analysis also shows what Saito and Rehmsmeier [33] recommend in their study: the PR-curve is more informative if we are dealing with imbalanced data in the population.

With that having said, we want to emphasize that we do not think that *Botometer* is a bad tool in general; we rather suggest that researchers should be aware of *Botometer*’s limitations when using the tool and care about validation with their own labeled data sets like Fernquist et al. [14], Cresci and colleagues [16], as well as Echeverrìa et al. [15] did in their studies. Our results are in line with this prior research as Botometer struggles with specific types of accounts, bots and humans, that are not covered by the training data [18]. We do think that *Botometer* can still be a useful tool for researchers, journalists as well as citizens. Just recently a new version (v4) of *Botometer* was released that addressed some aspects of our criticism [43] and has overall a better performance than prior versions of *Botometer*, such as the one (v3) analyzed in our study. Yet, major points of criticism that we identified here such as unclarity regarding what type of bot *Botometer* identifies, inadequate training data for other languages, and the potential vulnerability to temporal patterns still apply for *Botometer*’s new version. *Botometer* thus should, in this context, only be one step of a multi-step process that is built around human investigation of individual accounts [11]. While prior research has shown that humans in the case of crowdsourcing fail with more complex bots [16], we believe experienced researchers will still be able to identify bots with a combination of classifiers and manual forensic analysis. However, we believe that for computational social scientists working at scale, the uncertainty is too high. At least researchers should be aware of the data used as training data for the classifier that might only cover certain types of bots [15, 16, 18]. If researchers want to use *Botometer* for their datasets or even develop their own new classifiers we suggest certain steps going forward:

1. Manual classification: They should manually analyze a substantial sample of the classified bot and human accounts that were classified by the machine learning system. Researchers could then calculate the intercoder-reliability between the machine learning system’s classification and the human coder classification. While the percentage agreement is already an interesting indicator, we would highly recommend the use of a measurement like Krippendorff’s alpha [45] that takes into account chance agreement. They should also always manually validate, if possible, all the accounts that were classified as bots by the machine learning system if researchers want to make statements about the number of bots in a population. When a data set is too large for a complete manual analysis researchers should at least take an appropriate random sample for a manual classification. Furthermore, if the accounts in a data set are not homogenous (e.g., different languages, different topical context) researchers should use a stratified sampling strategy for manual post-hoc validation (see also point 4 below).
2. Time: Researchers should validate their results over time. As we’ve shown with *Botometer*, the scores are prone to changes over time. Consequently, researchers using *Botometer* or developing a new machine learning classifier should test their data set over time and look at the changes over time.
3. Language: Researchers should be aware of *Botometer*’s limitations (that are even stated by the creators of the tool) when it comes to classifying accounts that tweet in other languages. Hence, scholars will have to consider using other tools to check the number of bots in their data set; e.g., manual coding. Future machine learning systems should also consider different languages.
4. Data sharing: Researchers analyzing bots should share the user ids of the classified “bots” as the Twitter developer terms allows researchers to share the id of users. While the creators of *Botometer* share their lists of bots on their official homepage, social scientists using *Botometer* or similar machine learning systems in their studies often do not even share the ids of the accounts that got classified as bots.
5. Improvements: Going forward, researchers should support tools that are not based on black boxes but rather commit to the idea of open science. We should be able to work together on finding solutions and improving those solutions over time. This, then, would also allow us to deal more transparently with potential weaknesses.

Finally, there are other ways to identify bots on Twitter as Cresci [22] and Orabi et al. [20] show in their overviews. We believe it is important to consider the context as Bastos and Mercea [2], for example, have investigated suspicious patterns based on communication metrics as well as network analytics. And while this method might be more complicated and computationally expensive, it offers social scientists a way forward in identifying bots and understanding patterns of disinformation campaigns. Overall it is also important to broaden the perspective and focus more on political digital astroturfing [46] for which bots are just one potential dimension [47]. A potential solution could be to focus, as suggested in the literature, more on group behavior with unsupervised classifiers [22] in combination with a descriptive digital forensic analysis [11]. Still, our suggestions from above hold true even in this new scenario as Vargas and colleagues [44] show in the evaluation of such methods.

## Supporting information

S1 Fig Receiver operating characteristics curve for Botometer. The English score (left) and the English CAP (right). Average score over 3 months for each account.
(DOCX)

S2 Fig Distribution of the SD for single accounts plotted as groups. Left for the Botometer English score, right for the Botometer English CAP. Bandwith of 0.015 was used for the both.
(DOCX)

S3 Fig Changing score over time. Percentage of accounts (y-axis) that have at least once a score in the three months below as well as above the threshold for all thresholds between 0 and 1 in steps of 0.05. Left for the English score, right for the English CAP.
(DOCX)

S4 Fig CAP precision-recall curves for the resampled data sets. We consider the population baseline on Twitter (15% bots) for the English score (left) and the English CAP score (right). Black points indicate the precision and the recall for the Botometer English score 0.76 (left) and for the English CAP 0.25 (right). With the German politicians and bots for almost every threshold level the identified sample of bots has more humans than real bots (precision).
(DOCX)

S5 Fig Density plots for the different data sets. Left: Density plots for the different combined data sets in our analysis showing the distribution of Botometer’s English score. We used the resampled data sets with 15% bots and 85% humans with a total n = 100,000 for each data set. Right: Density plots for the human accounts data sets. Lines indicate the median, a bandwidth of 0.04 was used for all data sets.
(DOCX)
