# Supplementary material for: The False positive problem of automatic bot detection in social science research
Source: PLoS One. 2020 Oct 22;15(10):e0241045. doi: 10.1371/journal.pone.0241045 (PMC7580919; doi:10.1371/journal.pone.0241045)
Supplement: S1 Fig — The English score (left) and the English CAP (right). Average score over 3 months for each account. (DOCX) [file pone.0241045.s001.docx]

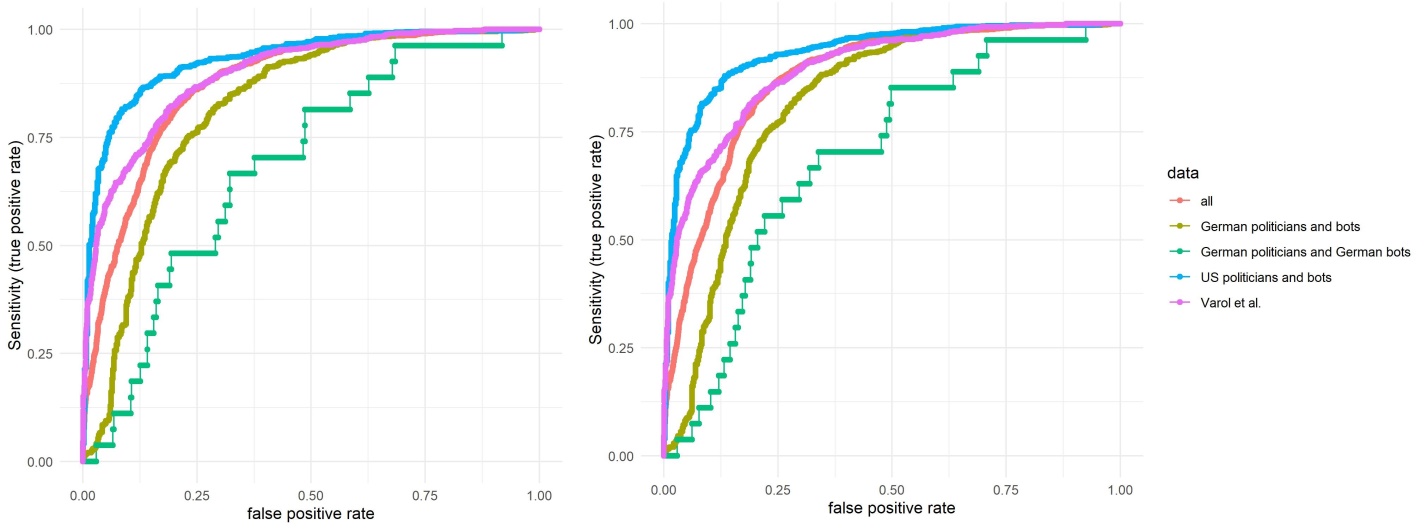


**S1 Fig. Receiver Operating Characteristics curve for Botometer.** The English score (left) and the English CAP (right). Average score over 3 months for each account.
