# Supplementary material for: The False positive problem of automatic bot detection in social science research
Source: PLoS One. 2020 Oct 22;15(10):e0241045. doi: 10.1371/journal.pone.0241045 (PMC7580919; doi:10.1371/journal.pone.0241045)
Supplement: S2 Fig — Left for the Botometer English score, right for the Botometer English CAP. Bandwith of 0.015 was used for the both. (DOCX) [file pone.0241045.s002.docx]

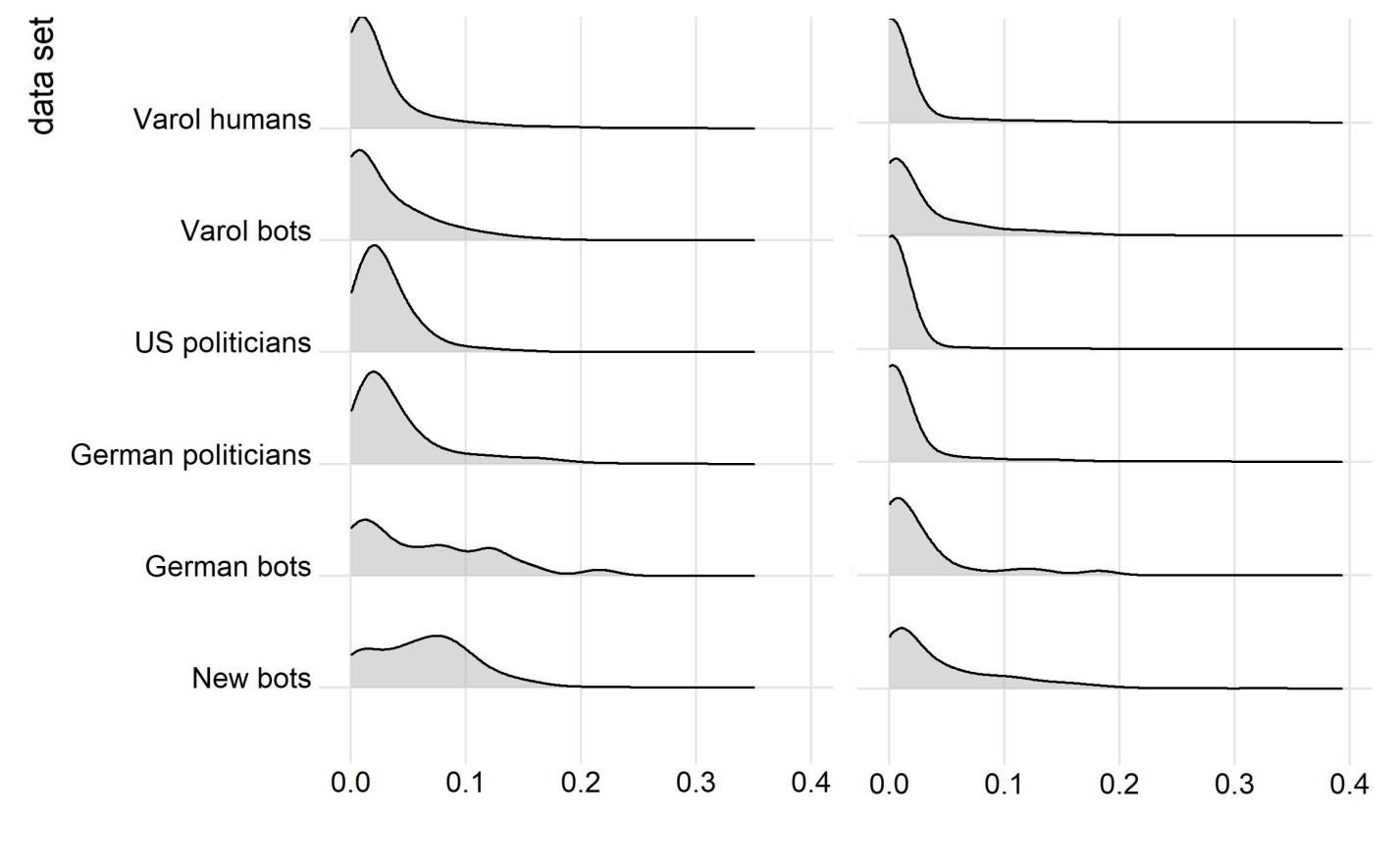


**S2 Fig. Distribution of the SD for single accounts plotted as groups**. Left for the Botometer English score, right for the Botometer English CAP. Bandwith of 0.015 was used for the both.
