# Supplementary material for: The False positive problem of automatic bot detection in social science research
Source: PLoS One. 2020 Oct 22;15(10):e0241045. doi: 10.1371/journal.pone.0241045 (PMC7580919; doi:10.1371/journal.pone.0241045)
Supplement: S3 Fig — Percentage of accounts (y-axis) that have at least once a score in the three months below as well as above the threshold for all thresholds between 0 and 1 in steps of 0.05. Left for the English score, right for the English CAP. (DOCX) [file pone.0241045.s003.docx]

**
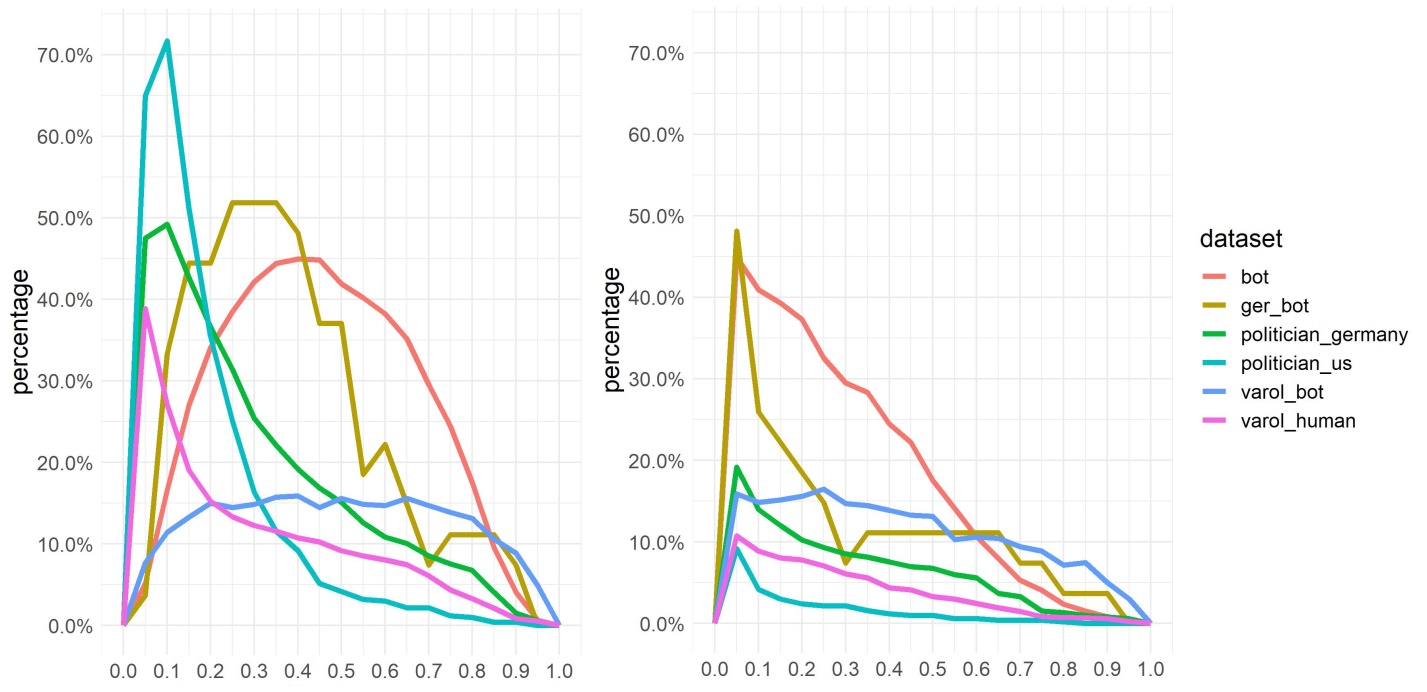
**

**S3 Fig. Changing score over time.** Percentage of accounts (y-axis) that have at least once a score in the three months below as well as above the threshold for all thresholds between 0 and 1 in steps of 0.05. Left for the English score, right for the English CAP.
