# Supplementary material for: The False positive problem of automatic bot detection in social science research
Source: PLoS One. 2020 Oct 22;15(10):e0241045. doi: 10.1371/journal.pone.0241045 (PMC7580919; doi:10.1371/journal.pone.0241045)
Supplement: S4 Fig — We consider the population baseline on Twitter (15% bots) for the English score (left) and the English CAP score (right). Black points indicate the precision and the recall for the Botometer English score 0.76 (left) and for the English CAP 0.25 (right). With the German politicians and bots for almost every threshold level the identified sample of bots has more humans than real bots (precision). (DOCX) [file pone.0241045.s004.docx]

**
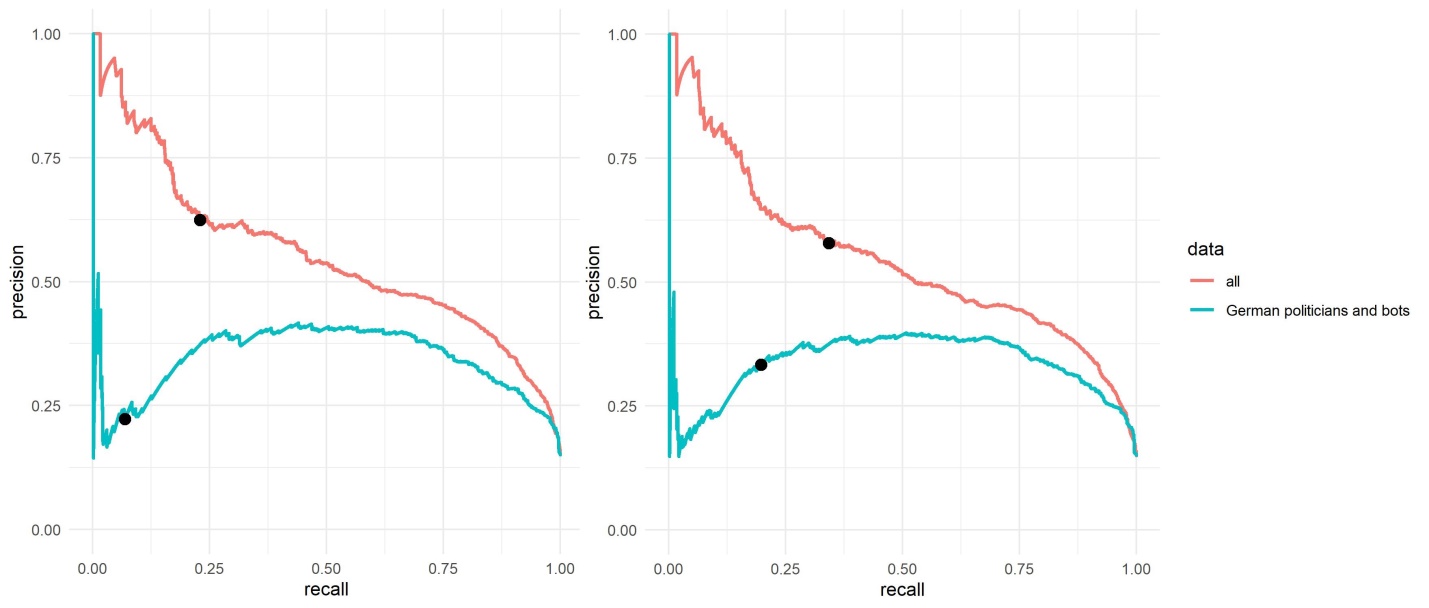
**

**S4 Fig. CAP precision-recall curves for the resampled data sets.** We consider the population baseline on Twitter (15% bots) for the English score (left) and the English CAP score (right). Black points indicate the precision and the recall for the Botometer English score 0.76 (left) and for the English CAP 0.25 (right). With the German politicians and bots for almost every threshold level the identified sample of bots has more humans than real bots (precision).
