# Supplementary material for: The False positive problem of automatic bot detection in social science research
Source: PLoS One. 2020 Oct 22;15(10):e0241045. doi: 10.1371/journal.pone.0241045 (PMC7580919; doi:10.1371/journal.pone.0241045)
Supplement: S5 Fig — Left: Density plots for the different combined data sets in our analysis showing the distribution of Botometer’s English score. We used the resampled data sets with 15% bots and 85% humans with a total n = 100,000 for each data set. Right: Density plots for the human accounts data sets. Lines indicate the median, a bandwidth of 0.04 was used for all data sets. (DOCX) [file pone.0241045.s005.docx]

**
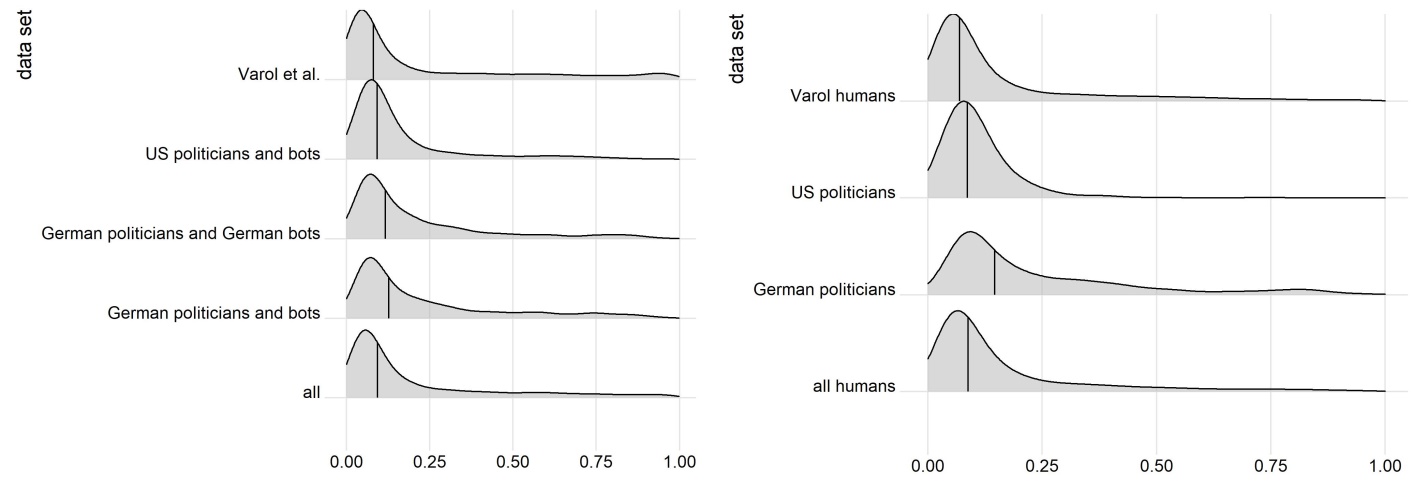
**

**S5 Fig. Density plots for the different data sets.** Left: Density plots for the different combined data sets in our analysis showing the distribution of Botometer’s English score. We used the resampled data sets with 15% bots and 85% humans with a total n=100,000 for each data set. Right: Density plots for the human accounts data sets. Lines indicate the median, a bandwidth of 0.04 was used for all data sets.
